# Production of antibodies with peptide-CpG-DNA-liposome complex without carriers

**DOI:** 10.1186/1471-2172-12-29

**Published:** 2011-05-18

**Authors:** Dongbum Kim, Sanghoon Kwon, Jae Won Rhee, Kwang Dong Kim, Young-Eun Kim, Cheung-Seog Park, Myeong Jun Choi, Jun-Gyo Suh, Doo-Sik Kim, Younghee Lee, Hyung-Joo Kwon

**Affiliations:** 1 Department of Microbiology, College of Medicine, Hallym University, Gangwon-do 200-702, Republic of Korea; 2 Center for Medical Science Research, College of Medicine, Hallym University, Gangwon-do 200-702, Republic of Korea; 3 Division of Applied Life Science (BK21 Program), PMBBRC, Gyeongsang National University, Jinju 660-701, Republic of Korea; 4 Department of Biochemistry, College of Natural Sciences, Chungbuk National University, Chungbuk 361-763, Republic of Korea; 5 Department of Microbiology, College of Medicine, Kyung Hee University, Seoul 130-701, Republic of Korea; 6 Korea Clinical Research Center Cp., Ltd, 899-6 Hogye-dong, Anyang, Gyeonggi-do 431-836, Republic of Korea; 7 Department of Medical Genetics, College of Medicine, Hallym University, Gangwon-do 200-702, Republic of Korea; 8 Department of Biochemistry, College of Science, Yonsei University, Seoul 120-749, Republic of Korea

## Abstract

**Background:**

The screening of peptide-based epitopes has been studied extensively for the purpose of developing therapeutic antibodies and prophylactic vaccines that can be potentially useful for treating cancer and infectious diseases such as influenza virus, malaria, hepatitis B, and HIV. To improve the efficacy of antibody production by epitope-based immunization, researchers evaluated liposomes as a means of delivering vaccines; they also formulated adjuvants such as flagella and CpG-DNA to enhance the magnitude of immune responses. Here, we provide a potent method for peptide-based epitope screening and antibody production without conventional carriers.

**Results:**

We present that a particular form of natural phosphodiester bond CpG-DNA encapsulated in a specific liposome complex (Lipoplex(O)) induces potent immunomodulatory activity in humans as well as in mice. Additionally, Lipoplex(O) enhances the production of IgG2a specific to antigenic protein in mice. Most importantly, immunization of mice with several peptides co-encapsulated with Lipoplex(O) without carriers significantly induces each peptide-specific IgG2a production in a TLR9-dependent manner. A peptide-specific monoclonal antibody produced against hepatocellular carcinoma-associated antigen has functional effects on the cancer cells.

**Conclusions:**

Our overall results show that Lipoplex(O) is a potent adjuvant and that complexes of peptide and Lipoplex(O) are extremely useful for B cell epitope screening and antibody production without carriers. Therefore, our strategy may be promptly used for the development of therapeutic antibodies by rapid screening of potent B cell epitopes.

## Background

Synthetic oligodeoxynucleotides (ODNs) and bacterial DNA containing unmethylated CpG dinucleotides flanked by specific base sequences (CpG-DNA) have significant immunomodulatory effects on B lymphocytes, macrophages, dendritic cells, and natural killer cells [1-4]. Experimental evidence suggests that CpG-DNA induces the regulation of Th1/Th2 immune responses, antigen-presenting cell activity, and immunoglobulin (Ig) isotype switching [5-7]. Therefore, CpG-DNA has gained attention for its potential use as an immune adjuvant and in therapeutics for allergic and infectious diseases [8,9].

Phosphorothioate-modified types of CpG-DNA (PS-ODN), which are resistant to nuclease activity and can be efficiently delivered into cells [10,11], have been utilized in clinical applications [9]. The immunomodulatory activities of PS-ODN are enhanced by liposome-encapsulation [12-14]. However, several studies have suggested that PS-ODN induces backbone-related side effects, such as transient splenomegaly [15], lymphoid follicle destruction [16], arthritis [17], and PS-ODN-specific IgM production [18] in PS-ODN-treated mice. Investigators consequently developed phosphodiester bond CpG-DNA (PO-ODN) as a natural counterpart of PS-ODN to induce optimal innate immune responses without severe side effects. In contrast to PS-ODN, the immunomodulatory effects of PO-ODN are found only in mouse cells and not in human cells [19]. However, induction of an effective immune response has been reported in human cells stimulated with PO-ODN and non-CpG-DNA encapsulated in cationic liposomes such as *N*-[1-(2,3-dioleoyloxy)propyl]-*N,N,N*-trimethylammonium methylsulfate (DOTAP) and lipofectin [20,21].

In previous studies, we screened natural PO-ODN with immunomodulatory activity from *Mycobacterium bovis *genomic DNA [22]. Our experimental analyses demonstrated that a potent PO-ODN, namely MB-ODN 4531(O), which contains three CpG motifs, has functional effects as a powerful adjuvant for the induction of Ag-driven Th1 responses without causing severe side effects in mice [18,22]. In this study, we compared the ability of MB-ODN 4531(O) encapsulated in several different liposomes to stimulate immune responses in human and mice cells, finding that MB-ODN 4531(O) encapsulated in a phosphatidyl-β-oleoyl-γ-palmitoyl ethanolamine (DOPE):cholesterol hemisuccinate (CHEMS) complex (Lipoplex(O)) was most potent in human as well as in mice. Furthermore, we extended the research to the selection of a synthetic peptide-based B cell epitope and revealed that complexes of several peptides and Lipoplex(O) without carriers significantly enhanced the each peptide-specific IgG production depending on TLR9. In this study, we identified a B cell epitope peptide from hepatocellular carcinoma (HCC)-specific transmembrane 4 superfamily member 5 (TM4SF5) protein [23] that potently induced epitope-specific antibodies. We also noticed that the monoclonal antibody produced by immunization with a complex consisting of antigenic peptide (TM4SF5R2-3) and Lipoplex(O) had functional effects on cells expressing the antigen. Our results suggest that the selection of B cell epitope can be facilitated by the delivery of the DOPE:CHEMS complex and by the adjuvant effect of MB-ODN 4531(O). Our strategy may be promptly used for the development of epitope-based peptide vaccines and production of therapeutic antibodies.

## Results

### Effects of CpG-DNA encapsulated in liposomes on IL-8 promoter activation

**Figure 1:**
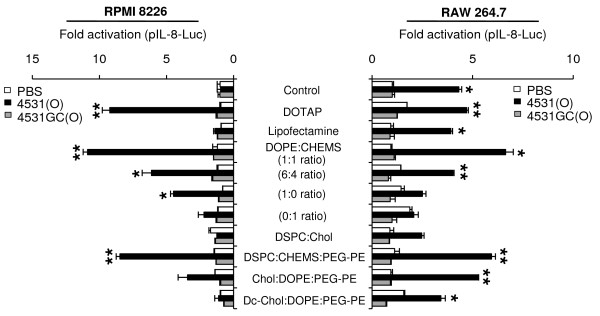
**Effect of CpG-DNA encapsulated in different liposome complexes on IL-8 promoter activation**. Human RPMI 8226 cells (left) and mouse RAW 264.7 cells (right) were transiently transfected with pIL-8-*Luc *for 24 h and stimulated with indicated liposomes or CpG-DNA (PO-ODN) encapsulated in the indicated liposome complexes for 12 h. Cultures were harvested and assayed for luciferase assay. The luciferase activity of the cells was measured in relative light units, and this value was normalized to *Renilla *activity. The results are represented as fold activation and compared to the PBS-treated control. Each bar represents Mean ± SD values of three experiments. *P < 0.05, **P < 0.01 (*vs *PBS control).

To identify the conditions driving the effective immunomodulatory activity of PO-ODN in humans, we encapsulated MB-ODN 4531(O) in several different liposomes and compared the abilities of the complexes to stimulate immune responses in human and mouse cells. First, we confirmed that IL-8 promoter was activated in human and mouse cells treated with CpG-DNA encapsulated in liposomes. When PO-ODN was encapsulated in a DOTAP, DOPE:CHEMS (1:1 ratio) complex, or 1,2-dioctadecanoyl-sn-glycero-3-phosphocholine (DSPC):CHEMS:phosphatidylethanolamine-poly(ethylene glycol) (PEG-PE) (6:4:0.3 ratio) complex, it activated the IL-8 promoter in human RPMI 8226 cells as well as in mouse RAW 264.7 cells. To confirm the significance of CpG motif of MB-ODN 4531(O), we synthesized MB-ODN 4531GC(O) which has GpC dinucleotides instead of CpG dinucleotides in the MB-ODN 4531(O) sequence. The luciferase activity was not enhanced by MB-ODN 4531GC(O) in the mouse and human cell lines, proving that the IL-8 promoter activation is CpG sequence-dependent (Figure 1). The highest activity was produced by PO-ODN encapsulated in DOPE:CHEMS (1:1 ratio) complex. Here, we named MB-ODN 4531(O) encapsulated in DOPE:CHEMS (1:1 ratio) complex as Lipoplex(O) (Table 1).

**Table 1:** Abbreviations used in this study.

Abbreviations	Descriptions
4531(O)	MB-ODN 4531(O)
4531GC(O)	MB-ODN 4531GC(O)
DOPE:CHEMS	Complex consisting of DOPE:CHEMS (1:1 ratio)
Lipoplex(O)	Complex of MB-ODN 4531(O) encapsulated with DOPE:CHEMS
DOPE:CHEMS + HEL (OVA or peptide)	Complex of HEL (OVA or peptide) encapsulated with DOPE:CHEMS
Lipoplex(O) + HEL (OVA or peptide)	Complex of HEL (OVA or peptide) and MB-ODN 4531(O) co-encapsulated with DOPE:CHEMS
4531(O) + HEL (OVA or peptide)	Mixture of MB-ODN 4531(O) and HEL (OVA or peptide)
LipoplexGC(O) + HEL (OVA or peptide)	Complex of HEL (OVA or peptide) and MB-ODN 4531GC(O) co-encapsulated with DOPE:CHEMS

### Enhanced intracellular uptake of CpG-DNA by encapsulation in a DOPE:CHEMS complex

**Figure 2:**
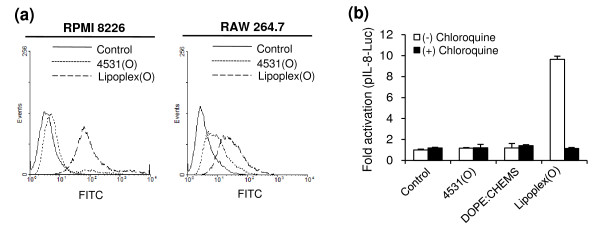
**Effect of CpG-DNA encapsulated in DOPE:CHEMS on intracellular uptake and endosomal localization of CpG-DNA**. (a) Effect of DOPE:CHEMS (1:1 ratio) encapsulation on uptake of CpG-DNA was analyzed by FACS. RPMI 8226 cells and RAW 264.7 cells were treated with FITC-labeled CpG-DNA or FITC-labeled CpG-DNA encapsulated in DOPE:CHEMS complex for 10 min. Intracellular uptake of CpG-DNA was measured by FACS analysis. 4531(O), FITC-labeled MB-ODN 4531(O); Lipoplex(O), FITC-labeled MB-ODN 4531(O) encapsulated in DOPE:CHEMS complex. (b) Involvement of endosomal acidification in IL-8 promoter activation induced by CpG-DNA encapsulated in DOPE:CHEMS complex was confirmed by chloroquine pretreatment. Luciferase activity in was measured in relative light units, and the value was normalized to *Renilla *activity. The results are represented in terms of fold activation and are compared to the PBS-treated control. Each bar represents Mean ± SD values obtained from three experiments. **P < 0.01 (*vs *PBS control).

Depending on the delivery vehicles, CpG-DNA can elicit enhanced intracellular uptake, endosomal localization, and biological activity of CpG-DNA [20]. To chase the process, we used FACS to analyze the intracellular uptake of FITC-labeled MB-ODN 4531(O). As shown in Figure 2a, the intracellular uptake of MB-ODN 4531(O) is enhanced markedly when the MB-ODN 4531(O) is encapsulated in a DOPE:CHEMS complex (Lipoplex(O)). TLR9-mediated cytokine production by CpG-DNA requires acidification and maturation of endosomes [24]. As shown in Figure 2b, pretreatment with chloroquine, an inhibitor of endosomal processing, inhibits the IL-8 promoter activation induced by Lipoplex(O). These results confirm that the efficacy of PO-ODN can be enhanced by encapsulation in a DOPE:CHEMS complex via the improved intracellular uptake of CpG-DNA and endosomal localization.

### Effects of CpG-DNA encapsulated in DOPE:CHEMS complex on cytokine production

**Figure 3:**
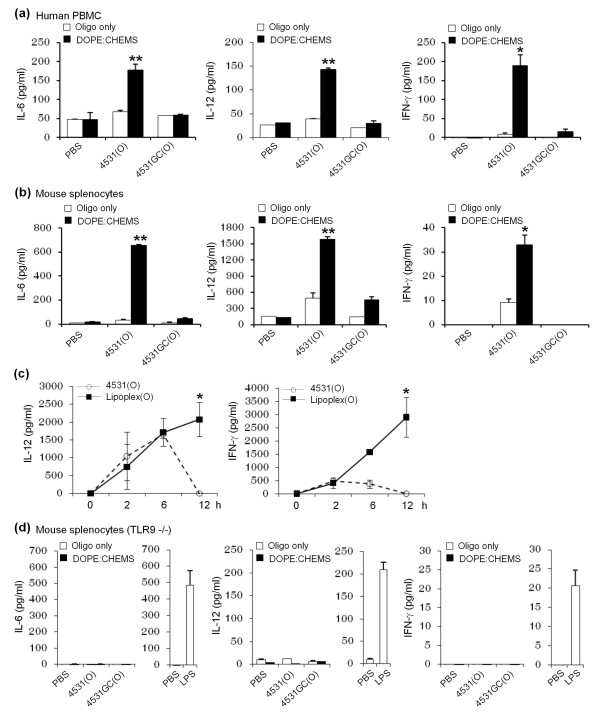
**Production of cytokines in hPBMC and mouse splenocytes by CpG-DNA encapsulated in DOPE:CHEMS complex**. The levels of IL-6, IL-12, and IFN-γ were measured by ELISA assay (a, b and d) or a Cytokine Bead Array kit (c). hPBMC (a), splenocytes from BALB/c mice (b), and splenocytes from BALB/c TLR9 knockout mice (TLR9-/-, n = 3/group) (d) were stimulated with LPS, free MB-ODN (oligo only) or MB-ODN encapsulated in a DOPE:CHEMS (1:1 ratio) complex for 24 h and the culture supernatants were harvested. The levels of IL-6, IL-12, and IFN-γ were measured with an ELISA assay. (c) BALB/c mice (n = 4/group) were injected i.p with MB-ODN 4531(O) or Lipoplex(O), and sera from the mice were harvested at the indicated times after injection. The concentration of IL-12p70 and IFN-γ in the serum was determined by using a Cytokine Bead Array kit. Each bar and graph represents Mean ± SD values obtained from three or four mice. These experiments were performed 2 times with similar results. *P < 0.05, **P < 0.01 (*vs *PBS control).

To determine the effects of Lipoplex (O) on cytokine production, we treated human peripheral blood mononuclear cells (PBMCs) or mouse splenocytes with 50 μg/ml of nonencapsulated CpG-DNA or Lipoplex(O) for 12 h. The CpG-DNA failed to induce the release of IL-6, IL-12, and IFN-γ in human PBMCs when treated as a free form. In contrast, Lipoplex(O) induced the release of IL-6, IL-12, and IFN-γ in a CpG sequence-dependent manner from human PBMCs and mouse splenocytes (Figure 3a and 3b). When MB-ODN 4531(O) or Lipoplex(O) was applied *in vivo*, Lipoplex(O) induced secretion of IL-12 and IFN-γ for much longer periods than MB-ODN 4531(O) did (Figure 3c). It may be related with the enhanced stability of MB-ODN 4531(O) *in vivo *when encapsulated in DOPE:CHEMS complex. As expected, the immunomodulatory activity of Lipoplex(O) was entirely TLR9-dependent; cytokine production was not found in TLR9-/- mice (Figure 3d). These results confirm that cytokine secretion was increased by Lipoplex(O) in a CpG sequence- and TLR9-dependent manner.

### Adjuvant activity of CpG-DNA encapsulated in DOPE:CHEMS complex

**Figure 4:**
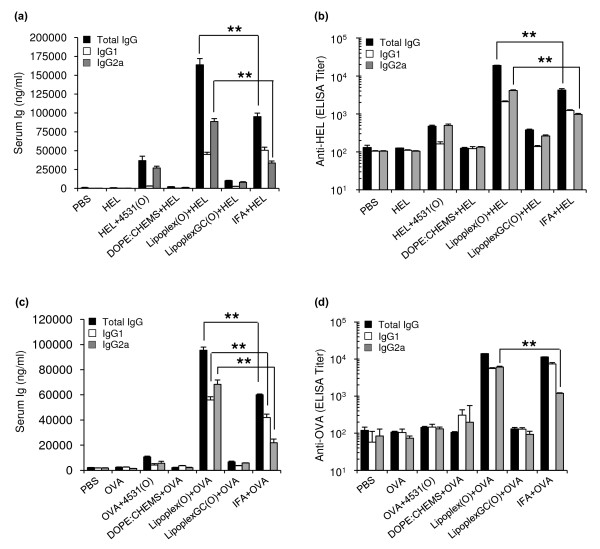
**Production of Th1-associated HEL (or OVA) antigen-specific IgG induced by CpG-DNA encapsulated in DOPE:CHEMS complex**. (a) Amounts of anti-HEL-specific total IgG and IgG isotypes. (b) Titers of anti-HEL-specific total IgG, anti-HEL-specific IgG1, and anti-HEL-specific IgG2a. (c) Amounts of anti-OVA-specific total IgG and IgG isotypes. (d) Titers of anti-OVA-specific total IgG, anti-OVA-specific IgG1, and anti-OVA-specific IgG2a. Three BALB/c mice were immunized with one of the following combinations: 50 μg of HEL (or OVA); 50 μg of MB-ODN 4531(O) and 50 μg of HEL (or OVA); 50 μg of HEL (or OVA) with DOPE:CHEMS; 50 μg of Lipoplex(O) and 50 μg of HEL (or OVA); or 50 μg of Lipoplex GC(O) and 50 μg of HEL; IFA and 50 μg of HEL (or OVA). The sera were collected, and the total IgG and IgG isotypes were assayed with an ELISA kit. Each bar represents Mean ± SD values obtained from three mice. This experiment was performed 3 times with similar results. **P < 0.01 (*vs *IFA + HEL (or OVA)).

To evaluate the adjuvant activity of CpG-DNA encapsulated in liposomes, we immunized BALB/c mice with different combinations of hen egg lysozyme (HEL), MB-ODN 4531(O), and DOPE:CHEMS complex (Figure 4). Immunization of BALB/c mice with HEL only or with HEL encapsulated in DOPE:CHEMS complex did not induce HEL-specific IgG. Immunization with a mixture of HEL and MB-ODN 4531(O), there was increased production of HEL-specific total IgG, which can be resulted from the adjuvant effect of MB-ODN 4531(O). Most importantly, immunization with a complex consisting of HEL and Lipoplex(O) (Lipoplex(O) + HEL) resulted in a markedly greater abundance of HEL-specific total IgG than other mice. Amount of HEL-specific total IgG induced by a complex of HEL and Lipoplex(O) was approximately four times higher than that induced by a mixture of HEL and MB-ODN 4531(O) (Figure 4a). Titers of HEL-specific total IgG also show similar results (Figure 4b). Next, we confirmed the IgG isotype of anti-HEL-specific antibodies. Remarkably, immunization with a complex of HEL and Lipoplex(O) or a mixture of HEL and MB-ODN 4531(O) induced more IgG2a than IgG1, indicating that MB-ODN 4531(O) affects isotype profile independently of encapsulation (Figure 4a). However, immunization with a mixture of HEL and incomplete Freund's adjuvant (IFA) induced higher level of IgG1 than IgG2a. Based on the result from the LipoplexGC(O) plus HEL, we confirmed that Lipoplex(O)-mediated HEL-specific antibody production is CpG sequence-dependent which is in accordance with the data in Figure 1. When we performed the same experiments with another antigen ovalbumin (OVA), Lipoplex(O) plus OVA appears to induce more IgG2a and hence a more balanced response than IFA plus OVA as shown in Figure 4c and 4d.

### Th1-dominated humoral immune response induced by peptide epitope and CpG-DNA encapsulated in DOPE:CHEMS complex

As the potent adjuvant effect of Lipoplex(O) was identified in Figure 4, we applied this system to the screening of B cell epitopes against tumor-specific antigen. Specifically, we aimed to screen B cell epitopes originated from human tetraspanin transmembrane 4 superfamily member 5 of human HCC (hTM4SF5) (GenBank: NP_003954) [23]. Six candidate peptide sequences were selected from extracellular domain of hTM4SF5 protein on the basis of their hydrophilicity, hydrophobicity, secondary structure, antigenicity index as described in "Methods" (Table 2).

**Table 2:** Candidate epitopes of hTM4SF5.

Proteins	Peptides	Sequences	Location	Length
	hTM4SF5R1	NGETSWTNTNHLSL	32-45	14
	hTM4SF5R2-1	RNGPRCLMNGEWGY	113-126	14
hTM4SF5	hTM4SF5R2-2	GEWGYHFEDTAGAY	122-135	14
	hTM4SF5R2-3	NRTLWDRCEAPPRV	138-151	14
	hTM4SF5R2-4	WDRCEAPPRVVPWN	142-155	14
	hTM4SF5R2-5	GAYLLNRTLWDRCEA	133-147	15

**Figure 5:**
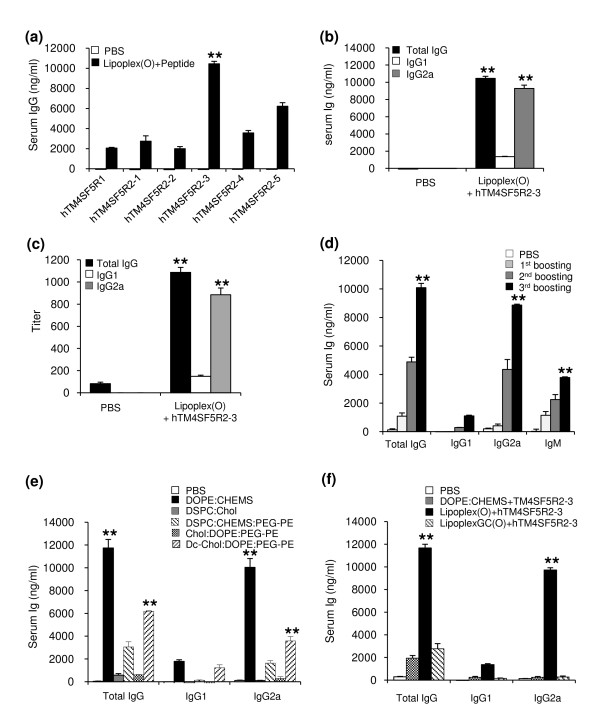
**Selection of epitopes and production of epitope-specific IgG by a complex of peptide and Lipoplex(O)**. (a-c) Selection of B cell epitopes from hTM4SF5 protein by immunization with complexes consisting of each predicted hTM4SF5 peptide and Lipoplex(O). BALB/c mice (n = 3/group) were immunized with complexes consisting of each peptide (hTM4SF5R1, hTM4SF5R2-1, hTM4SF5R2-2, hTM4SF5R2-3, hTM4SF5R2-4, and hTM4SF5R2-5) and Lipoplex(O) (Lipoplex(O) + peptide). (a) Amounts of anti-each peptide-specific total IgG. (b) Amounts of anti-hTM4SF5R2-3 peptide-specific IgG isotypes. (c) Titers of anti-hTM4SF5R2-3 peptide-specific IgG isotypes. (d) The kinetics of IgG production. BALB/c mice (n = 3/group) were immunized with the complex of hTM4SF5R2-3 peptide and Lipoplex(O). BALB/c mice were injected i.p. with hTM4SF5R2-3 peptide and Lipoplex(O) complex. The sera were collected one day before each injection and 10 days after final injection, and an ELISA kit was used to assay amounts of the peptide-specific total IgG, IgG1, IgG2a and IgM were assayed with an ELISA kit. (e) Induction of DOPE:CHEMS encapsulation-dependent peptide-specific IgG production. BALB/c mice (n = 5/group) were immunized with 50 μg of hTM4SF5R2-3 peptide and 50 μg of MB-ODN 4531(O) coencapsulated in a DOPE:CHEMS (1:1 ratio), DSPC:Chol (45:55 ratio), DSPC:CHEMS:PEG-PE (6:4:0.3 ratio), Chol:DOPE:PEG-PE (4:6:0.06 ratio), or Dc-Chol:DOPE:PEG-PE (4:6:0.06 ratio) complex. The sera were collected, and amounts of anti-hTM4SF5R2-3 peptide-specific total IgG were assayed with an ELISA kit. (f) Effect of GC dinucleotide and phosphorothioate backbone modification. BALB/c mice (n = 5/group) were immunized with 50 μg of hTM4SF5R2-3 peptide and one of the following complexes (50 μg): Lipoplex(O), or LipoplexGC(O). The sera were collected, and amounts of peptide-specific total IgG were assayed with an ELISA kit. The results are expressed as a Mean ± SD of three or five mice. These experiments were performed 3 times with similar results. **P < 0.01 (*vs *PBS control).

**Figure 6:**
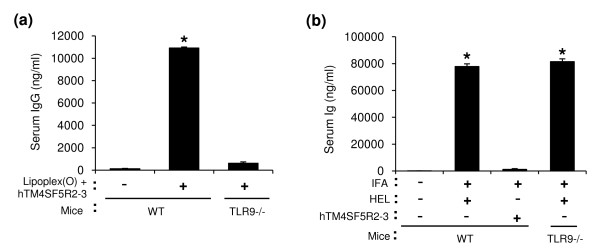
**Induction of TLR9-dependent epitope-specific IgG production by a complex of peptide and Lipoplex(O)**. BALB/c mice (WT) and BALB/c TLR9 knockout mice (TLR9-/-) (n = 3/group) were immunized with hTM4SF5R2-3 peptide (50 μg/mouse) and Lipoplex(O) complex (Lipoplex(O) + TM4SF5R2-3) (a), IFA with 50 μg of HEL (b), or IFA with hTM4SF5R2-3 peptide (b). The sera were collected, and amounts of the peptide-specific total IgG were assayed with an ELISA kit. The results are expressed as a Mean ± SD of three mice. This experiment was performed 2 times with similar results. *P < 0.05 (*vs *control mice).

First, we screened the B cell epitopes of hTM4SF5 protein. We analyzed the amounts of IgG in the serum of BALB/c mice injected i.p. with complexes of each predicted hTM4SF5 peptide and Lipoplex(O). As shown in Figure 5a, the production of peptide-specific IgG was most enhanced by a complex consisting of hTM4SF5R2-3 peptide and Lipoplex(O). Anti-hTM4SF5R2-3-specific antibodies were mostly composed of IgG2a (Figure 5b and 5c). We evaluated the kinetics of IgG production in immunized mice in response to the complex of hTM4SF5R2-3 peptide and Lipoplex(O). The mice produced larger amounts of peptide-specific IgG (IgG2a) during the secondary and tertiary responses (Figure 5d). When the mice were immunized with hTM4SF5R2-3 peptide and MB-ODN 4531(O) co-encapsulated in other liposomes, the production of peptide-specific IgG was much lower, which demonstrates the superior effect of Lipoplex(O) (Figure 5e). However, MB-ODN 4531GC(O) containing a reversal sequence of the CG dinucleotide lost its ability to stimulate production of IgG (Figure 5f). IgG production by hTM4SF5R2-3 peptide and Lipoplex(O) was dependent on TLR9, whereas IgG production by antigen and IFA was not (Figure 6). These results indicate that the complex of hTM4SF5R2-3 peptide and Lipoplex(O) without carriers significantly enhanced production of peptide-specific IgG2a in a CpG sequence-dependent and TLR9-dependent manner.

### Functional effect of anti-hTM4SF5 monoclonal antibody on hepatocellular carcinoma cells *in vitro*

**Figure 7:**
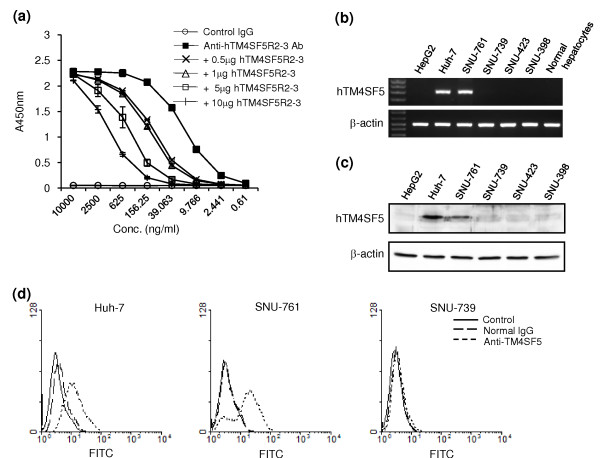
**Detection of TM4SF5 by anti-hTM4SF5 monoclonal antibody produced by hTM4SF5R2-3 peptide and Lipoplex(O) complex**. (a) Reactivity of anti-hTM4SF5R2-3 monoclonal antibody with hTM4SF5R2-3 peptide. hTM4SF5R2-3 peptide was immobilized on the plate, and competitive ELISA was performed using increasing amounts of soluble hTM4SF5R2-3 peptide. (b) Expression of hTM4SF5 mRNA in the human hepatocarcinoma cell lines. The expression level of hTM4SF5 mRNA was analyzed by RT-PCR with cDNAs from the indicated cell lines and normal hepatocytes. (c-d) Detection of hTM4SF5 by anti-hTM4SF5R2-3 peptide monoclonal antibody in human hepatocarcinoma cell lines. (c) The expression of hTM4SF5 protein was analyzed by immunoblotting with anti-hTM4SF5R2-3 peptide mAb in human hepatocarcinoma cell lines. (d) The hepatocarcinoma cell lines were stained with anti-hTM4SF5R2-3 peptide monoclonal antibody and then FITC-conjugated antibody to anti-mouse IgG, and analyzed with a FACScan flow cytometer. Normal mouse IgG was used as a control.

**Figure 8:**
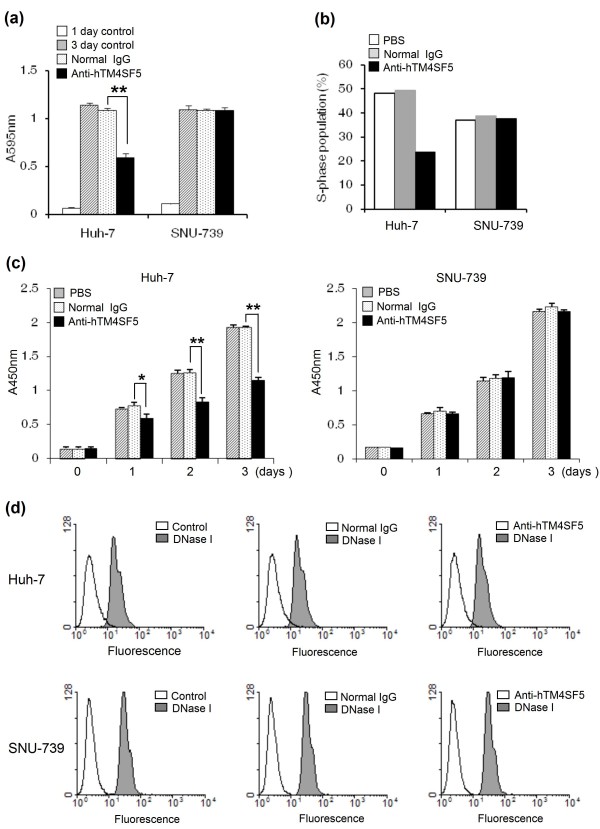
**Functional effects of anti-hTM4SF5 monoclonal antibody produced by immunization with hTM4SF5R2-3 peptide and Lipoplex(O) complex**. (a) Effect of anti-hTM4SF5R2-3 peptide monoclonal antibody on cell growth. Cell growth was measured by MTT assay. Each bar is expressed as a Mean + SD of three experiments. **P < 0.01 (*vs *normal IgG control). (b) Effect of anti-hTM4SF5R2-3 peptide monoclonal antibody on cell cycle. The cells were stained with propidium iodide in PBS containing RNase and analyzed by flow cytometry. (c) Effect of anti-hTM4SF5R2-3 peptide monoclonal antibody on proliferation. The DNA synthesis activity was monitored by BrdU incorporation assay. Each bar is expressed as a Mean ± SD of three experiments. *P < 0.05, **P < 0.01 (*vs *normal IgG control). (d) Effect of anti-hTM4SF5R2-3 peptide monoclonal antibody on apoptosis. FACSCalibur flow cytometer was used to analyze cells containing fluorescein-labeled double strand DNA breaks. As a positive control, the cells were treated with DNase I prior to analysis. These experiments were performed three times with similar results.

The expression of TM4SF5 induced uncontrolled growth of human HCC cells via loss of contact inhibition [23]. To evaluate the functional effects of IgG produced by immunization with the complex of hTM4SF5R2-3 peptide and Lipoplex(O), we screened monoclonal antibody against hTM4SF5R2-3 peptide and confirmed its specificity by competition ELISA using hTM4SF5R2-3 peptide (Figure 7a). We then analyzed the expression of hTM4SF5 in human HCC cell lines and evaluated the monoclonal antibody-mediated hTM4SF5 targeting in the cells. The expression of hTM4SF5 was detected in Huh-7 and SNU-761 cells at the mRNA by RT-PCR (Figure 7b) and protein levels by immunoblotting and FACS analysis with the hTM4SF5-specific monoclonal antibody (Figure 7c and 7d). To investigate the effect of the anti-hTM4SF5R2-3 peptide monoclonal antibody on cell growth, we performed MTT assay. The growth of Huh-7 cells expressing hTM4SF5 was markedly delayed by antibody treatment. However, there was no significant reduction in the growth of SNU-739 cells, which did not express hTM4SF5 (Figure 8a). The population of cells in S phase was also reduced in Huh-7 cells but not SNU-739 cells (Figure 8b) To further investigate the effects of the anti-hTM4SF5R2-3 peptide antibody on DNA synthesis, the BrdU incorporation assay was performed. As shown in Figure 8c, the rate of DNA synthesis in Huh-7 cells treated with anti-hTM4SF5R2-3 peptide monoclonal antibody was weakly affected one day after treatment and further suppressed in three days by approximately 40% compared to normal IgG treated-Huh-7 cells (Figure 8c). In contrast, there was no effect in SNU-739 cells. Next, we investigated whether the anti-hTM4SF5R2-3 peptide antibody induces apoptotic response of Huh-7 cells. As shown in Figure 8d, the treatment of anti-hTM4SF5R2-3 peptide antibody did not lead to an increase in the number of apoptotic cells containing double-stranded DNA breaks in Huh-7 cells or SNU-739 cells. Therefore, we conclude that the anti-hTM4SF5R2-3 peptide antibody has anti-proliferative effect rather than pro-apoptotic effect in Huh-7 cells. These results confirm that the monoclonal antibody produced by immunization with hTM4SF5R2-3 peptide and Lipoplex(O) complex was useful for hTM4SF5 protein detection and antibody-mediated hTM4SF5 targeting in HCC cells.

## Discussion

The application of CpG-DNA to therapeutics can be optimized via sequence rearrangement and backbone modification [25,26]. The recognition of CpG-DNA by TLR9 varies from mice to human immune cells due to a 24% difference at the amino acid level between the TLR9 molecules of mice and humans [8,19]. PS-ODN stimulates the human and mouse immune systems in several ways: efficient uptake into cells, endosomal acidification and maturation, and enhanced recognition by TLR9 in endosomes and lysosomes [24]. However, PO-ODN, which exhibits immunomodulatory activity in mice, shows poor activity in hPBMCs [8,27]. Recently, the cationic liposome DOTAP was used as a vehicle for delivery; it was found to improve the uptake of PO-ODN as well as the production of IFN-α in hPBMCs [20]. Here, we examined the effects of liposome-encapsulated PO-ODN on cytokine promoter activation and cytokine production in mouse and human cells. After stimulation with PO-ODN encapsulated in liposomes such as DOTAP, DOPE:CHEMS complex, and DSPC:CHEMS:PEG-PE complex, the IL-8 promoter was significantly activated in human and mouse cells and maximum activity was seen with PO-ODN encapsulated in DOPE:CHEMS complex (Lipoplex(O)) (Figure 1).

Recent studies by several investigators have shown that CpG-DNA has functional effects as a Th1-responsive adjuvant and that its potent adjuvant effects are enhanced by liposome encapsulation [12,14]. In this report, we found that Lipoplex(O) surpassed IFA in terms of the potency of antigen-specific IgG production and Th1-type immune responses in mice (Figure 4): higher titers and more IgG2a. This outstanding adjuvant effect of Lipoplex(O) in mice is very interesting and suggests that Lipoplex(O) could have a promising adjuvant effect in humans. The potency of Lipoplex(O) seems to be derived from the improved intracellular uptake of CpG-DNA and endosomal localization (Figure 2), and enhanced or prolonged production of cytokines such as IL-6, IL-12, and IFN-γ (Figure 3).

**Figure 9:**
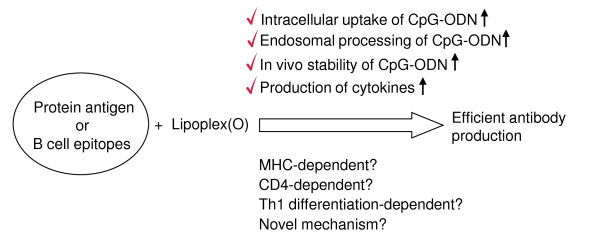
**Possible mechanisms involved in the adjuvant effect of Lipoplex(O)**. The activity of Lipoplex(O) confirmed in this study are checked above the arrow. The issues to be determined in the future are shown below the arrow.

The screening of synthetic peptide-based epitopes has been studied extensively for the purpose of developing therapeutic antibodies and vaccines that have potential as prophylactics for cancer and infectious diseases such as influenza virus, malaria, hepatitis B, and HIV [28-30]. Although peptide vaccines have been actively studied in various animal models, their efficacy in the treatment of humans is limited. To improve the efficacy of peptide vaccines, researchers have evaluated liposomes as a means of delivering vaccines [13,31-33]. Adjuvants such as flagella [34] and CpG-DNA [13] have also been formulated in order to enhance the magnitude of the immune responses. In this study, we performed peptide-based epitope screening and used complex of synthetic peptide and Lipoplex(O) without carriers to produce antibodies that act against hTM4SF5 in human HCC. We successfully screened a potent B cell epitope (hTM4SF5R2-3 peptide) from hTM4SF5 (Figure 5). We found that Lipoplex(O) surpassed IFA and other forms of liposome-encapsulated CpG-DNA in terms of the potency of peptide-specific IgG production and Th1-type immune responses in mice (Figure 5 and 6). Therefore, our data offer a powerful tool for practical use: Lipoplex(O) is a potentially applicable universal adjuvant for peptide-based epitope screening and antibody production, and it obviates the need for other adjuvants and carriers. However, further study is needed to investigate whether or not IgG production induced by a complex of B cell epitope and Lipoplex(O) is dependent on MHC type, CD4^+ ^cells and Th1 differentiation and if a novel mechanism is involved (Figure 9).

Examination of the IgG isotype levels demonstrates that the peptide and Lipoplex(O) complex induced higher levels of IgG2a than IgG1, indicating a predominant Th1 response. It is well-known that differential induction of Th1-type or Th2-type immune response by a vaccine is important for protective immunity in specified infectious diseases. For example, several studies have reported that formalin-inactivated human respiratory syncytial virus (RSV) vaccine was not effective because of its ability to induce an allergy-like Th2 immune response against the virus infection [35-37]. Therefore, our formulation may be applied as an effective adjuvant for peptide-based RSV B cell epitope screening and effective RSV vaccine: the formulation may decrease susceptibility to RSV and induce Th1-biased or balanced Th1/Th2 adaptive immune response leading to the production of neutralizing antibodies.

This study suggest that Lipoplex(O) is a potent adjuvant and that complexes of epitope and Lipoplex(O) without carriers are extremely useful for B cell epitope screening and peptide-specific antibody production. As our novel vaccine strategy using Lipoplex(O) and synthetic peptide does not require protein antigen production and carrier conjugation, it is cost effective and enables rapid application. For example, TM4SF5 protein has gained attention as a target for HCC therapy since it is overexpressed in HCC [23]. TM4SF5 is also important in HCC formation by inducing morphological elongation, epithelial-mesenchymal transition, abnormal cell growth in multilayers *in vitro*, and tumor formation *in vivo*. But there was no report regarding epitope-specific monoclonal antibody against TM4SF5 yet. As indicated in a detailed study, we successfully produced monoclonal antibody without production of recombinant protein. The anti-hTM4SF5R2-3 peptide-specific antibody can detect native protein and induce functional changes in cells expressing TM4SF5 protein, which suggests possible application in therapeutics (Figure 7 and 8). We are currently investigating whether or not anti-hTM4SF5R2-3 peptide-specific antibody can reduce HCC tumor formation in a xenograft mouse model after Huh-7 cells have been injected. We applied our strategy to monomeric peptide and produced antibody recognizing native protein in this study. Whether our technology can also be applied to conformationally dependent epitopes or poorly immunogenic peptides is to be determined in the future.

## Conclusions

**Figure 10:**
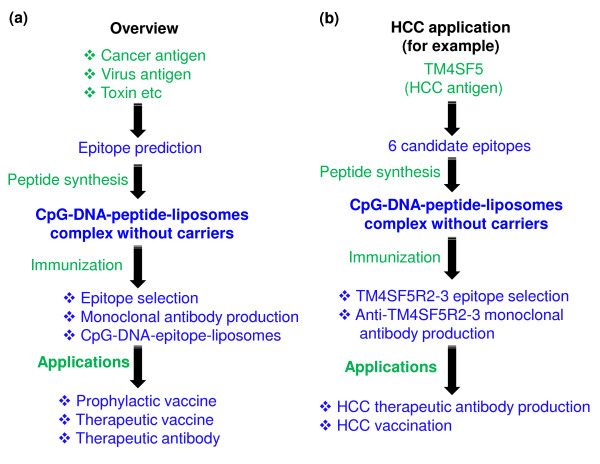
**Schematic diagrams summarizing the experimental process**. (a) General overview of experiments and possible applications. (b) Specific procedure performed in this work and possible applications.

Our overall results show that Lipoplex(O) is a potent adjuvant and that complexes of epitope and Lipoplex(O) without carriers are extremely useful for B cell epitope screening and peptide-specific antibody production. Therefore, our strategy may be promptly used for the development of therapeutic antibodies by rapid screening of potent B cell epitopes (Figure 10).

## Methods

### ODNs and reagents

ODNs were obtained from Samchully Pharm (Seoul, Korea) and GenoTech (Daejeon, Korea). MB-ODN 4531 consisted of 20 bases containing three CpG motifs (underlined): AGCAGCGTTCGTGTCGGCCT. The sequence of MB-ODN 4531 used in this study was phosphodiester bond (O). MB-ODN 4531GC(O) is a derivative of MB-ODN 4531(O) with one of its CG sequences reversed to GC (underlined): AGCAGGCTTCGTGTCGGCCT. When necessary, biotin or fluorescent tags were conjugated to the 3' end of each ODN. The endotoxin content of the ODNs was less than 1 ng/mg of ODN when measured by *Limulus amebocyte *assay (Whittaker Bioproducts, Walkersville, MD, USA). To block the endosomal localization of CpG-DNA, we pretreated the cells with chloroquine (10 μM) for 1 h before stimulation with CpG-DNA or liposome-encapsulated CpG-DNA.

### Preparation of protein (or peptide) and CpG-DNA co-encapsulated in liposome complexes

The liposomes CHEMS, Chol, DOPE, and DSPC were purchased from Sigma-Aldrich (St. Louis, MO, USA). 3β-(N-(N',N'-dimethylaminoethane)-carbamoyl)cholesterol (DC-Chol) and PEG-PE were obtained from Avanti-Polar Lipids (Alabaster, AL, USA). Complexes of CpG-DNA and protein (or peptide) with DOTAP (Roche, Indianapolis, IN, USA), lipofectamine (Invitrogen, Carlsbad, CA, USA) or lipofectin (Invitrogen) were prepared in accordance with the manufacturer's specifications. Liposome complexes consisting of protein (or peptide) and CpG-DNA co-encapsulated with DOPE:CHEMS, DSPC:Chol (55:45 ratio), DSPC:CHEMS:PEG-PE (6:4:0.3 ratio), Chol:DOPE:PEG-PE (4:6:0.06 ratio) or Dc-Chol:DOPE:PEG-PE (4:6:0.06 ratio) were prepared as reported previously [38] with minor modifications. Briefly, DOPE and CHEMS were mixed in ethanol at a molar ratio of 1:1, or as indicated in the individual experiments, evaporated with nitrogen gas to make a solvent-free lipid film, and resuspended in a mixture containing equal volumes of water-soluble CpG-DNA and protein (or peptide), followed by vigorous stirring at room temperature for 30 min. After adjusting the pH to 7.0, the Lipoplex solution was slightly sonicated for 30 s with a sonicator. We then filtered the solution with a 0.22 μm filter and freeze-thawed it three times with liquid nitrogen.

### Transfection and luciferase assays

Transfection and luciferase assays were performed as previously described [26]. For transfection with an IL-8 promoter-reporter construct, we used FuGENE 6 Transfection Reagent (Roche). RPMI 8226 cells (human B cell line) and RAW 264.7 cells (mouse macrophage cell line) were treated with CpG-DNA (5 μg/ml) or CpG-DNA encapsulated in liposomes for 12 h, or as indicated in the individual experiments.

### Uptake of CpG-DNAs

RPMI 8226 cells and RAW 264.7 cells were plated in 24-well plates for 24 h and then treated with FITC-labeled CpG-DNA or FITC-labeled CpG-DNA encapsulated in DOPE:CHEMS complex for 10 min. Intracellular uptake of CpG-DNA was measured by FACS analysis with a FACSCalibur cytometer (BD Biosciences, San Diego, CA, USA) as reported previously [39].

### Measurement of cytokines

To measure cytokines, CpG-DNA (5 μg/ml) or CpG-DNA (5 μg/ml) encapsulated in DOPE:CHEMS complex was added to splenocytes or hPBMCs, followed by incubation at 37°C with 5% CO_2 _for 24 h. The cytokine levels in the culture supernatant were then measured using commercially available ELISA kits (R&D Systems, Minneapolis, MN, USA). The amount of cytokines in the sera was quantified using a cytokine bead array kit for mouse inflammatory cytokines (CBA; BD Biosciences) and a FACSCalibur cytometer equipped with CellQuestPro and CBA software.

### Selection and synthesis of B cell epitope peptides

The putative B cell epitope peptides of hTM4SF5 were selected on the basis of hydrophilicity values from Parker *et al *[40], surface accessibility values from Emini *et al *[41], β-turn region values from Chou and Fasman [42], and antigenicity index from Kolaskar and Tongaonkar [43]. The parameters were averaged over six amino acid residues and the regions above the threshold value 1.0 were chosen for each prediction factor (http://tools.immuneepitpoe.org/main/index.html). To screen B cell epitope, we selected 6 candidates from the putative B cell epitope peptides, which are located in the extracellular domain of hTM4SF5 (Table 2). The peptides were synthesized using an automated peptide synthesizer (Peptron III-R24, Peptron, Daejeon, Korea). The peptides were purified by reverse-phase HPLC (Prominence HPLC, Shimadzu Corp., Tokyo, Japan) to purity greater than 90%. The peptide was identified using a mass spectrometer (HP 1100 Series LC/MSD, Hewlett-Packard, Roseville, CA, USA).

### Mice and immunization

Mice were maintained under specific-pathogen-free conditions. We purchased four-week-old male BALB/c (H-2^b^) mice from Central Lab. Animal, Inc. (Seoul, Korea), BALB/c TLR9 knockout mice from Oriental Bioservice, Inc. (Kyoto, Japan). Our animal studies were approved by the Institutional Animal Care and Use Committee of Hallym University (Hallym 2009-47). On three occasions at 10 day intervals, the mice were injected intraperitoneally (i.p.) with 50 μg of HEL, OVA, or peptides (50 μg) supplemented with either 50 μg of CpG-DNA or CpG-DNA encapsulated in liposomes.

### Antigen-specific Ig ELISA assay

Mouse sera were obtained by orbital bleeding before each injection as well as by sacrifice 10 days after final injection and then stored at -70°C. To measure the amounts of total IgG, IgG1, and IgG2a, 96-well immunoplates were coated with 5 μg/ml of each protein or peptide and then blocked with 0.05% of Tween-20 in PBS (PBST) containing 1% BSA. The sera were diluted to 1:450 with PBST and added to the wells of each plate. To measure the titers of total IgG, IgG1 and IgG2a, the sera were added to the top row of each plate, and serial 1:3 dilutions in PBST were then placed into subsequent rows. The plates were incubated for 2 h at room temperature and washed with PBST. Then, rat anti-mouse biotinylated secondary antibodies (total IgG, IgG1, or IgG2a) (BD Biosciences) were added to the wells and incubated for 1 h, followed by addition of streptavidin conjugated with horseradish peroxidase for 30 min. A colorimetric assay was developed with a TMB substrate solution (Kirkegaard and Perry Laboratories, Gaithersburg, MD, USA), and we used a Spectra Max 250 microplate reader (Molecular Devices, Sunnyvale, CA, USA) to measure the absorbance at 450 nm. Antibody titers were defined as the reciprocal serum dilution yielding half-maximal signal.

### Production of mouse anti-hTM4SF5 monoclonal antibody

On three occasions at 10 day intervals, BALB/c mice were injected i.p. with hTM4SF5R2-3 peptide (50 μg) and MB-ODN 4531(O) (50 μg) co-encapsulated in DOPE:CHEMS complex. In accordance with standard hybridoma technique, we then screened hybridoma cells that produce anti-hTM4SF5R2-3 peptide-specific monoclonal antibody [44]. Anti-hTM4SF5R2-3 peptide monoclonal antibody (IgG2a) was purified from the ascites fluid by protein A column chromatography.

### Reactivity of mouse anti-hTM4SF5 monoclonal antibody: Competitive ELISA assay

To measure the titers of anti-TM4SF5R2-3 peptide monoclonal antibody, we coated 96-well immunoplates with 10 μg/ml of human TM4SF5R2-3 peptides and then blocked them with PBST containing 1% BSA. The antibodies were added to the top row of each plate, and serial 1:4 dilutions in PBST were then placed into subsequent rows. For competition assay, diluted antibodies were preincubated with the indicated amounts of each peptide for 30 min and then added to the wells of each plate. The plates were incubated for 2 h at room temperature, washed with PBST, and then incubated with anti-IgG antibody conjugated with horseradish peroxidase for 2 h. The colorimetric assay was developed with a TMB substrate solution (Kirkegaard and Perry Laboratories), and we used a Spectra Max 250 microplate reader (Molecular Devices, Sunnyvale, CA, USA) to measure the absorbance at 450 nm.

### Cell culture

The human B cell line RPMI 8226, mouse macrophage cell line RAW 264.7, and the human hepatoma cell lines Huh-7 and HepG2 were obtained from ATCC. The human hepatocellular cell lines SNU-398, SNU-423, SNU-739, and SNU-761 were obtained from the Korean Cell Line Bank (Seoul, Korea). The cells were maintained in RPMI 1640 medium containing 10% FBS. Human normal hepatocyte cells (Promo Cell, Heidelberg, Germany) were maintained as the vendor suggested. All cells were cultured at 37°C in an atmosphere of 95% air and 5% CO_2_.

### Detection of TM4SF5 expression

To analyze hTM4SF5 expression, we performed RT-PCR, immunoblotting, and FACS analysis. Total RNAs were extracted with an RNeasy Mini Kit (Qiagen, Germantown, MD, USA), and the cDNA was generated as described previously [26]. The standard PCR reaction was performed for 25 cycles with the following primer sets: human β-actin, 5'-GGGTCAGAAGGATTCCTATG-3' and 5'-CCTTAATGTCACGCACGATTT-3' (500 bp); hTM4SF5, 5'-AGCTTGCAAGTCTGGCTCAT-3' and 5'-GCTGGATCCCACACAGTACT-3' (408 bp); The expression of hTM4SF5 protein was confirmed by immunoblotting and FACS analysis with purified anti-hTM4SF5 monoclonal antibody.

### MTT assay

The growth of cells treated with anti-hTM4SF5 monoclonal antibody (5 μg/ml) was determined by MTT assay with 3-(4,5-dimethylthiazole-2-yl)-2,5-diphenyl tetrazolium bromide (MTT, Sigma-Aldrich) solution as previously described [45].

### BrdU proliferation assay

To assess the cell proliferation after treatment with anti-hTM4SF5 monoclonal antibody, Huh-7 and SNU-739 cells were seeded into a 96-well microplate at a density of 10^5 ^cells/well. The cells were treated with anti-hTM4SF5 monoclonal antibody (5 μg/ml) for the indicated time periods. Subsequently, cells were fixed, washed, and incubated with primary and secondary antibodies according to the instruction of CycLex BrdU Cellular ELISA Kit (MBL International, Woburn, USA). The immune complexes were developed with a substrate solution, and we used a Spectra Max 250 microplate reader (Molecular Devices, Sunnyvale, CA, USA) to measure the absorbance at 450 nm.

### Cell cycle analysis

To analyze DNA content, we incubated the cells in a solution of propidium iodide (20 μg/ml) in PBS containing RNase (200 μg/ml). The cells were stained at room temperature for 30 min and analyzed using FACSCalibur cytometer (BD Biosciences).

### TUNEL assay

We performed terminal deoxynucleotidyl transferase-mediated dUTP nick-end labeling (TUNEL) assay in accordance with the manufacturer's specifications (Promega, Madison, WI, USA). Briefly, Huh-7 and SNU-739 cells were placed in a complete medium for 24 h before being treated with anti-hTM4SF5 monoclonal antibody (5 μg/ml) for 3 days. The cells were fixed with 4% paraformaldehyde and permeabilized with 0.2% Triton X-100 for 5 min at room temperature, and end-labeled with TdT using the DeadEnd™ Fluorometric TUNEL system. The DNase I treatment served as a control of TUNEL-positive cells. After washing with PBS-T, cells were observed with FACSCalibur cytometer (BD Biosciences).

## Abbreviations

CpG-DNA: synthetic DNA containing immunostimulatory CpG motifs; CHEMS: cholesterol hemisuccinate; DOPE: phosphatidyl-β-oleoyl-γ-palmitoyl ethanolamine; Lipoplex(O): MB-ODN 4531(O) encapsulated in DOPE:CHEMS (1:1 ratio) complex; PO-ODN: natural phosphodiester CpG-DNA; hTM4SF5: human tetraspanin transmembrane 4 superfamily member 5.

## Authors' contributions

HJK and YL designed the research, supervised the experiments, and wrote the paper. DK and BKP performed the molecular research and epitope screening, analyzed the function of the epitope and Lipoplex(O) complex, and prepared the figures. JWR and MJC prepared complexes of the epitope and Lipoplex(O) and analyzed the data. SK contributed to the hTM4SF5 experiments. CSP and YEK carried out monoclonal antibody production and analysis. KDK, JGS and DSK performed the knockout mouse experiment, cytokines measurement, and participated in paper writing. All authors read and approved the final manuscript.
